# Patterns of resident health workforce turnover and retention in remote communities of the Northern Territory of Australia, 2013–2015

**DOI:** 10.1186/s12960-017-0229-9

**Published:** 2017-08-15

**Authors:** Deborah J Russell, Yuejen Zhao, Steven Guthridge, Mark Ramjan, Michael P Jones, John S Humphreys, John Wakerman

**Affiliations:** 1 Monash Rural Health, Monash University, PO Box 666, Bendigo, Victoria 3552 Australia; 2 Department of Health, PO Box 40596, Darwin, NT 0800 Australia; 3 Faculty of Human Sciences, Macquarie University, North Ryde, NSW 2109 Australia; 4 Monash Rural Health, Monash University, PO Box 91, Strathdale, Victoria 3550 Australia; 5 Flinders Northern Territory, School of Medicine, Flinders University, PO Box U362, Casuarina, NT 0815 Australia

**Keywords:** Remote health, Rural workforce, Health workforce, Fly-in/fly-out, Rural health services, Aboriginal, Aboriginal health practitioner, Remote area nurse, Turnover, Retention

## Abstract

**Background:**

The geographical maldistribution of the health workforce is a persisting global issue linked to inequitable access to health services and poorer health outcomes for rural and remote populations. In the Northern Territory (NT), anecdotal reports suggest that the primary care workforce in remote Aboriginal communities is characterised by high turnover, low stability and high use of temporary staffing; however, there is a lack of reliable information to guide workforce policy improvements. This study quantifies current turnover and retention in remote NT communities and investigates correlations between turnover and retention metrics and health service/community characteristics.

**Methods:**

This study used the NT Department of Health 2013–2015 payroll and financial datasets for resident health workforce in 53 remote primary care clinics. Main outcome measures include annual turnover rates, annual stability rates, 12-month survival probabilities and median survival.

**Results:**

At any time point, the clinics had a median of 2.0 nurses, 0.6 Aboriginal health practitioners (AHPs), 2.2 other employees and 0.4 additional agency-employed nurses.

Mean annual turnover rates for nurses and AHPs combined were extremely high, irrespective of whether turnover was defined as no longer working in *any* remote clinic (66%) or no longer working at a *specific* remote clinic (128%). Stability rates were low, and only 20% of nurses and AHPs remain working at a *specific* remote clinic 12 months after commencing. Half left within 4 months.

Nurse and AHP turnover correlated with other workforce measures. However, there was little correlation between most workforce metrics and health service characteristics.

**Conclusions:**

NT Government-funded remote clinics are small, experience very high staff turnover and make considerable use of agency nurses. These staffing patterns, also found in remote settings elsewhere in Australia and globally, not only incur higher direct costs for service provision—and therefore may compromise long-term sustainability—but also are almost certainly contributing to sub-optimal continuity of care, compromised health outcomes and poorer levels of staff safety. To address these deficiencies, it is imperative that investments in implementing, adequately resourcing and evaluating staffing models which stabilise the remote primary care workforce occur as a matter of priority.

## Background

Geographical maldistribution of the health workforce is a persisting global issue that has been linked with the inability of rural and remote populations to gain equitable access to health services and consequent poorer health outcomes [1]. Maldistribution results in absolute and relative shortages of rural primary care workers in many countries, irrespective of the country’s wealth [2–7]. In Australia, the 2013 Review of Australian Government Health Workforce Programs found that the most significant primary care workforce issue was the distribution of health workers [8]. Addressing inequities relating to the maldistribution of health workers requires workforce planners to have a good understanding of health worker transitions into and out of rural and remote areas. This, in turn, requires measurement of these transitions and monitoring changes over time, so that the effectiveness of any strategies devised to optimise health worker recruitment, turnover and retention can be developed and assessed.

Recent research suggests that no single workforce metric assessing turnover or retention is sufficient, as each is likely to have considerable limitations if used in isolation [9]. Instead, a suite of measures provides more comprehensive information about both the health workers who are leaving rural and remote communities and, perhaps more importantly, those who are staying [10, 11]. While the health workforce literature identifies many different indicators of turnover and of retention [12], key metrics relevant to the Australian rural and remote health workforce context include annual turnover rates, stability rates, survival probabilities and median survival [9]. A second component of workforce mobility is the level at which turnover or retention outcomes are determined and reported. This level has been variously defined, for example, retention in a practice, retention in a community, and retention in a larger health organisation [13]. Data from the United States of America indicate annual health service turnover rates for hospital-based nurses of up to 20% [14, 15]. Other comparative research, also from the USA, suggests that annual hospital staff turnover rates of 4–12% are low, 12–21% medium and 22–44% high [16]. Australian research reports average annual organisational turnover of nurses from the Northern Territory Government (NTG) Department of Health (DoH) of 35% and organisational turnover of permanent Queensland Health nurses of 20%, with much higher turnover rates and lower stability rates experienced by nurses working in smaller more remote health services [17, 18].

Published Northern Territory (NT) reports are now 10 or more years old, and considerable changes have occurred in the ensuing period, particularly with regard to the Australian Government’s NT Emergency Response (NTER) which was initiated in 2007 [19]. While many changes occurred in the targeted NT remote Aboriginal communities as a result of NTER, one key feature impacting on the health workforce in remote communities was the extra financial resources available, leading to increased use of short-term visiting health workers. The Remote Area Health Corps (RAHC), for example, was established in 2008 and received Australian Government funding to recruit metropolitan-based health professionals for periods of service of 3 to 12 weeks [20]. It has been suggested, however, that high levels of short-term visiting health workers in NT Aboriginal communities causes frustration for experienced permanent staff and may impact negatively on long-term service sustainability, quality of care and, ultimately, health outcomes. However, the extent to which quantitative evidence supports such assertions has not yet been rigorously examined [21, 22].

Additionally, in 2002, the Aboriginal and Torres Strait Islander Workforce National Strategic Framework proposed changes affecting the registration requirements, professional recognition and role of Aboriginal health workers (AHWs). In the same year, completion of Certificate 4 competency-based training became a requirement for newly registered AHWs in NT and for existing AHWs with Certificate 3 level qualifications who wished to progress to a higher career level. Numbers of AHWs registered in the NT fluctuated between approximately 250 and 340 between 2004 and 2010, a decline from registrations in excess of 400 in the late twentieth century [23]. In July 2012, national registration for Aboriginal and Torres Strait Islander health practitioners (hereafter referred to, using the NT term, as Aboriginal health practitioners, or AHPs) was introduced, replacing the previous classification of AHWs. In 2012, 2013 and 2014, there were 219, 216 and 201 registered AHPs in the NT respectively, representing a further decline in AHP numbers [24]. How these recent reductions in numbers of AHPs registered in NT have manifested in remote NT Aboriginal communities, in terms of supply, turnover and stability of AHPs employed in a permanent capacity by NTG DoH is not known.

Other gaps in knowledge include current turnover rates, stability rates and survival probabilities for nurses and for AHPs; correlation between key workforce metrics in these remote contexts; and whether there are differences in turnover rates, stability rates and health worker survival patterns amongst the different remote communities and the extent to which differences are related to observable community characteristics such as geographical remoteness, community population size and health service size. Finally, we do not know to what extent use of casual and agency nurse staffing in remote communities is associated with turnover and retention of permanent employees and hence with workforce sustainability in remote communities. These are important evidence gaps that limit the ability of workforce planners and policymakers to understand and address sub-optimal health service performance related to high turnover and poor retention of health workers in remote communities.

The aims of this paper, therefore, are threefold. Firstly, to describe current (2013–2015) health workforce turnover and retention patterns for nurses and AHPs living and providing clinical services in government health services in remote NT communities. Secondly, to investigate how key workforce metrics correlate with each other. Thirdly, to investigate how key workforce metrics correlate with health service characteristics, including community remoteness and isolation measures, health service population catchment, and health service size.

## Methods

### Study setting

This study is set in the remote NT of Australia, covering an area of approximately 1.3 million km^2^. The NT is sparsely settled. Of the population of approximately 244,000 people, 27% identify as Aboriginal. Most (80%) NT Aboriginal people live in remote towns and communities [25]. During the study period, the NTG DoH provided health care services in 53 remote NT communities, while non-government organisations (Aboriginal Controlled Community Health Organisations) provided health care services in a further 28 remote communities. Primary care delivery in remote NT communities is generally provided by resident Remote Area Nurses and Midwives (hereafter referred to as nurses) and AHPs with professional support provided by telehealth and scheduled intermittent visits from medical and allied health practitioners. Two health services in very small remote communities did not have nurses, and 11 health services did not have AHPs; however, there were no other systematic differences in health service characteristics. Primary care in remote communities was supported by secondary care available in five public NT hospitals. The focus of this study was on the resident clinical staff (nurses and AHPs) in remote NTG-operated health services.

### Data

Two separate data sources were used. The first was the Personnel Information and Payroll Systems (PIPS) data from NTG DoH (3 January 2013 to 30 December 2015). This provides comprehensive, individual-level, de-identified information on all nurses and AHPs paid directly through the NTG DoH payroll in any of the 53 remote clinics. Some nurses recruited through a nurse employment agency are employed on temporary or casual NTG DoH contracts and are captured by the PIPS dataset. Other agency nurses are paid directly by an agency and are therefore not included in PIPS data. These agency-employed nurses, however, are identifiable through labour hire costs in NTG Government Accounting System (GAS). GAS expenditures on agency nurse labour hire costs were used to derive the aggregated full-time equivalent (FTE) agency-employed nurses working in remote health services using the standard NTG DoH formula of agency labour expenses divided by twice the departmental annual average nurse personnel cost [26].

### Analysis

In this study, an exit was primarily defined at a health service level (primary turnover profile), that is, when an employee left one of the 53 specific health clinics for a period of more than 12 weeks.

A secondary definition of an exit, at a remote health level (secondary turnover profile), is also reported, when an employee ceased working in remote health, that is, no longer worked in any of the 53 remote health clinics. In this instance, remote inter-clinic moves were ignored.

Measures of health worker supply were number of unique employees, average annual headcounts, average FTE, and agency-employed nurse FTE ratios, defined as follows:

1. Total number of unique employees (sum of individuals employed in each year)
2. Average annual headcounts (average number of individuals employed in each pay period in each year)
3. Average FTE (average FTE employed in each pay period in each year)
4. Agency-employed nurse FTE ratio

$$ =\frac{\mathrm{average}\  \mathrm{agency}\  \mathrm{employed}\  \mathrm{nurse}\ \mathrm{FTE}\ }{\mathrm{sum}\ \mathrm{of}\  \mathrm{nurse}\ \mathrm{FTE}\ \mathrm{on}\ \mathrm{organisational}\  \mathrm{chart}\ } $$

The key turnover and retention metrics were averaged over 3 years and based on headcounts (except where specified as FTE):

1. The turnover measure was Annual turnover rates (%)

$$ =\frac{\mathrm{total}\  \mathrm{number}\  \mathrm{of}\  \mathrm{exits}\  \mathrm{in}\ 12\ \mathrm{month}\  \mathrm{period}\ }{\mathrm{average}\  \mathrm{number}\  \mathrm{of}\  \mathrm{employees}\kern0.5em \mathrm{in}\ 12\ \mathrm{month}\  \mathrm{period}}\times 100 $$

The retention measures were

2. Annual stability rates (%)

$$ =\frac{\mathrm{Number}\  \mathrm{of}\  \mathrm{employees}\ \mathrm{at}\ \mathrm{start}\  \mathrm{of}\  \mathrm{year}\ \mathrm{who}\ \mathrm{remain}\  \mathrm{employed}\ 12\ \mathrm{months}\  \mathrm{later}}{\mathrm{Number}\  \mathrm{of}\  \mathrm{employees}\ \mathrm{at}\ \mathrm{start}\  \mathrm{of}\  \mathrm{year}}\times 100 $$

3. Experienced nurses or AHPs

$$ ={\displaystyle \begin{array}{c}\hfill \mathrm{Number}\  \mathrm{of}\ \mathrm{pay}\ \mathrm{per}\mathrm{iods}\ \mathrm{per}\ \mathrm{calendar}\  \mathrm{year}\  \mathrm{with}\ \mathrm{at}\ \mathrm{least}\ \mathrm{one}\ \mathrm{nurse}\  \mathrm{or}\ \mathrm{AHP}\ \hfill \\ {}\hfill \mathrm{who}\ \mathrm{has}\ \mathrm{been}\ \mathrm{at}\ \mathrm{that}\  \mathrm{health}\  \mathrm{service}\ \mathrm{for}\ 2\ \mathrm{or}\  \mathrm{more}\  \mathrm{year}\mathrm{s}\  \mathrm{continuously}\hfill \end{array}} $$

4. Survival probability after 12 months (nurses and AHPs)

$$ =\frac{\mathrm{Number}\  \mathrm{remaining}\  \mathrm{employed}\  \mathrm{beyond}\ 12\ \mathrm{months}\  \mathrm{after}\  \mathrm{commencing}\ \mathrm{a}\mathrm{t}\ \mathrm{a}\ \mathrm{remote}\  \mathrm{health}\  \mathrm{service}\ }{\mathrm{Number}\ \mathrm{a}\mathrm{t}\ \mathrm{risk}\  \mathrm{of}\  \mathrm{exiting}\ \mathrm{a}\ \mathrm{remote}\  \mathrm{health}\  \mathrm{service}} $$

5. Median survival time in years (nurses and AHPs)

$$ ={\displaystyle \begin{array}{c}\hfill \mathrm{Time}\  \mathrm{from}\  \mathrm{commencing}\ \mathrm{a}\mathrm{t}\ \mathrm{a}\ \mathrm{remote}\  \mathrm{health}\  \mathrm{service}\ \mathrm{a}\mathrm{t}\ \mathrm{which}\  \mathrm{the}\  \mathrm{probability}\  \mathrm{of}\  \mathrm{remaining}\ \hfill \\ {}\hfill \mathrm{employed}\ \mathrm{a}\mathrm{t}\ \mathrm{t}\mathrm{he}\  \mathrm{health}\  \mathrm{service}\  \mathrm{equal}\mathrm{s}\  \mathrm{the}\  \mathrm{probability}\  \mathrm{of}\  \mathrm{having}\  \mathrm{exited}\  \mathrm{which}\  \mathrm{is}\  \mathrm{equal}\ \mathrm{t}\mathrm{o}\ 0.5\hfill \end{array}} $$

Summary statistics for each of the key metrics were analysed by community population size and whether the communities were predominantly Aboriginal or not. Aboriginal community population size was distributed into four categories (<200, 200–349, 350–799, ≥800). Since there were only seven predominantly non-Aboriginal communities, it was not appropriate to stratify non-Aboriginal communities by population size. Community population size estimates were based on 30 June 2012 Australian Bureau of Statistics Estimated Resident Populations. 2011 Australian Bureau of Statistics Census data for Indigenous Locations were used to determine whether a community was predominantly (>50%) Aboriginal or not. 2012 ABS Estimated Resident Population data were very strongly (*r* = 0.97) correlated with 2014 health service records of catchment populations which clinic staff maintain on the number of currently active patients. Distances to the nearest hospital and to Darwin or Alice Springs (whichever was closer) were measured using Google Maps straight line distances in kilometres. The 2015–2016 NTG DoH Top End Health Service and Central Australia Health Service organisational charts were used to determine the number of FTE nurse and AHP positions at each remote health service. Summary statistics were reported as means with 95% confidence intervals or medians with interquartile ranges (IQR). Pearson correlations were used with a significance test to explore associations between key workforce metrics and also between key workforce metrics and health service characteristics.

Ethics approval was received from the Human Research Ethics Committee of the NTG DoH and Menzies School of Health Research (2015-2363).

## Results

In total, the number of unique nurses, AHPs and other staff members on the payroll for the 53 clinics in the 3-year study period were 470, 93 and 583 respectively. The average annual headcounts for nurses, AHPs and other employees were 272, 67 and 314 respectively. The total FTE for the 53 clinics was 68.7 and 23.8 for nurses and AHPs respectively. In any pay period, the median number of nurses per clinic was 1.97 (IQR 1.38, 3.29), AHPs per clinic was 0.60 (IQR 0.04, 1.27) and other employees per clinic was 2.20 (IQR 0.85, 3.85). The median number of nurse, AHP and other position types shown on 2015–2016 organisational charts for these remote health services was 2.0 (IQR 2.0, 4.0), 1.0 (IQR 1.0, 4.0), and 2.5 (IQR 1.7, 4.6) respectively.

The median straight line distance to the nearest hospital of these health services was 205 km (IQR 143, 248), while the median distance to Alice Springs or Darwin was 247 km (IQR 180, 390). The median population catchment for the health services was 460 (IQR 236, 798).

Overall, 347 nurse and AHP individuals ceased providing care in any remote health service (secondary turnover profile) during the 2013–2015 period. This was an annual turnover rate of 65.8% (95%CI 58.3, 74.1) for nurses and AHPs combined, with 74.4% (95%CI 65.3, 84.7) for nurses and 37.8% (95%CI 27.7, 51.6) for AHPs.

The mean overall annual turnover rate at the clinic level (primary turnover profile) for nurses and AHPs combined was estimated as 128% (95% CI 114, 144) (Table 1). Annual stability rates averaged 55.3% (95% CI 49.6, 61.6) for nurses and AHPs. The mean probability that nurses and AHPs stay at least 12 months is 0.20 (95% CI 0.16, 0.24), and within 0.34 (95% CI 0.27, 0.42) years of commencement, half the nurses and AHPs have left. Point estimates of the 12-month survival probability and median survival of AHPs were longer than for nurses, although this difference was not statistically significant (Tables 2 and 3). Average annual stability rates, however, were significantly higher for AHPs at 76.3% (95% CI 63.5, 91.7) compared with 48.3% (95% CI 42.3, 55.2) for nurses.

**Table 1:** Summary of nurse and Aboriginal health practitioner workforce metrics, by community population size and type

Population size category of remote community	Number of clinics	Mean annual nurse and AHP turnover (%) (95% CI)	Mean nurse and AHP annual stability (%) (95% CI)	Mean number of pay periods per year with an experienced nurse or AHP (95% CI)^a^	Mean survival probability for nurses and AHPs after 12 months (95% CI)	Average survival time for nurses and AHPs in years (95% CI)
<200 predominantly Aboriginal	7	120 (68, 209)	61.9 (37.6, 100.0)	9.7 (8.4, 11.1)	0.12 (0.03, 0.29)	0.38 (0.08, 0.88)
200–349 predominantly Aboriginal	12	172 (128, 232)	54.9 (40.5, 74.4)	17.0 (15.7, 18.4)	0.10 (0.04, 0.18)	0.19 (0.11, 0.31)
350–799 predominantly Aboriginal	16	149 (120, 185)	53.6 (43.5, 66.0)	18.6 (17.4, 19.8)	0.17 (0.11, 0.24)	0.23 (0.15, 0.31)
≥800 predominantly Aboriginal	11	116 (97, 138)	54.5 (46.4, 64.1)	23.3 (21.7, 25.0)	0.25 (0.19, 0.31)	0.46 (0.38, 0.54)
Predominantly non-Aboriginal	7	82 (56, 120)	60.8 (44.0, 83.9)	17.7 (16.0, 19.6)	0.24 (0.12, 0.38)	0.31 (0.11, 0.69)
Total	53	128 (114, 144)	55.3 (49.6, 61.6)	17.9 (17.3, 18.6)	0.20 (0.16, 0.24)	0.34 (0.27, 0.42)

Turnover and stability calculations done at an aggregate level for each category of population size

*AHP* Aboriginal health practitioners, *CI* confidence interval

^a^Poisson distribution assumed

**Table 2:** Summary of nurse workforce metrics, by community population size and type

Population size category of remote community	Number of clinics	Mean annual nurse turnover (%) (95% CI)	Mean nurse annual stability^a^ (%) (95% CI)	Mean number of pay periods per year with a nurse who has remained for 2 or more years (95% CI)^c^	Mean survival probability^b^ for nurses after 12 months (95%CI)	Average survival time for nurses in years (95% CI)	Mean agency-employed nurse FTE ratio
<200 predominantly Aboriginal	5	156 (76, 318)	57.9 (27.1, 100.0)	2.4 (1.8, 3.2)	0.14 (0.02, 0.36)	0.69 (0.04, 0.92)	0.04 (0.00, 1.68)
200–349 predominantly Aboriginal	12	202 (145, 281)	45.1 (30.9, 65.6)	8.3 (7.4, 9.3)	0.11 (0.05, 0.19)	0.19 (0.08, 0.31)	0.14 (0.04, 0.43)
350–799 predominantly Aboriginal	16	175 (136, 224)	47.2 (36.2, 61.5)	14.6 (13.5, 15.7)	0.13 (0.07, 0.19)	0.19 (0.15, 0.27)	0.16 (0.07, 0.35)
≥800 predominantly Aboriginal	11	130 (107, 158)	46.1 (37.5, 56.5)	18.9 (17.5, 20.5)	0.24 (0.18, 0.31)	0.42 (0.34, 0.54)	0.17 (0.10, 0.31)
predominantly non-Aboriginal	7	75 (50, 112)	59.1 (42.1, 83.1)	16.1 (14.4, 17.9)	0.27 (0.13, 0.42)	0.50 (0.15, 0.77)	0.10 (0.02, 0.46)
Total for nurses	51	148 (126, 163)	48.3 (42.3, 55.2)	12.6 (12.1, 13.2)	0.19 (0.15, 0.23)	0.31 (0.23, 0.38)	0.15 (0.10, 0.23)

Turnover and stability calculations done at an aggregate level for each category of population size

*CI* confidence interval, *FTE* full-time equivalent

^a^Annual stability rates were not estimated for two health services (both <200 population size category)

^b^Survival probability after 12 months was inestimable for one health service (<200 population size category)

^c^Poisson distribution assumed

**Table 3:** Summary of Aboriginal health practitioner workforce metrics, by community population size and type

Population size category of remote community	Number of clinics	Mean annual AHP turnover (%) (95% CI)	Mean AHP annual stability (%) (95% CI)	Mean number of pay periods per year with an AHP who has remained for 2 or more years (95% CI)^a^	Mean survival probability for AHPs after 12 months (95% CI)	Average survival^b^ time for AHPs in years (95% CI)
<200 predominantly Aboriginal	6	75.7 (29.7, 193.4)	75.0 (37.9, 100.0)	8.0 (6.8, 9.3)	0.09 (0.00, 0.35)	0.31 (0.08, 0.88)
200–349 predominantly Aboriginal	7	66.5 (29.4, 150.4)	87.1 (51.4, 100.0)	9.7 (8.7, 10.8)	0.00 (−, −)	0.23 (0.15, −)
350–799 predominantly Aboriginal	14	93.1 (58.6, 147.9)	73.6 (52.9, 100.0)	8.8 (8.0, 9.7)	0.45 (0.23, 0.64)	0.77 (0.23, 1.92)
≥800 predominantly Aboriginal	11	65.8 (42.6, 101.7)	75.6 (57.7, 99.1)	17.8 (16.3, 19.3)	0.30 (0.12, 0.50)	0.46 (0.15, 1.03)
Predominantly non-Aboriginal	4	169.0 (53.0, 539.1)	77.8 (28.4, 100.0)	3.7 (2.9, 4.6)	0.00 (−, −)	0.04 (0.04, −)
Total for AHPs	42	79.4 (60.5, 104.2)	76.3 (63.5, 91.7)	10.1 (9.6, 10.6)	0.27 (0.16, 0.39)	0.46 (0.23, 0.84)

Turnover and stability calculations done at an aggregate level for each category of population size

*AHP* Aboriginal health practitioners, *CI* confidence interval, *FTE* full-time equivalent

^a^Poisson distribution assumed (and the superscript in the cell with mean number of pay periods)

^b^Median survival inestimable for seven remote health services (two services in <200, three services in 200–349, one in 350–799, one in ≥ 800 population size categories)

Remote NT health services had high levels of agency-employed nurse use. Of the 51 health services that had nurse positions, agency-employed nurses provided a median of 0.40 FTE per clinic (IQR 0.26, 0.58). On average, for every 1.0 FTE nurse position on organisational charts, there were 0.15 FTE (95% CI 0.10, 0.23) agency-employed nurses.

Annual turnover rates for nurses and AHPs combined were significantly correlated with other workforce metrics, though not with agency-employed nurse FTE ratios (Table 4).

**Table 4:** Correlations between different workforce metrics for nurses and Aboriginal health practitioners, 2013–2015 data

	Annual nurse and AHP turnover	Nurse and AHP annual stability	Number of pay periods per year with a nurse or AHP who has remained for 2 or more years	Survival probability after 12 months	Median survival time	Agency-employed nurse FTE ratio
Annual nurse and AHP turnover	1					
Nurse and AHP annual stability	− 0.3103*	1				
Number of pay periods per year with a nurse or AHP who has remained for 2 or more years	− 0.3542**	0.5427**	1			
Survival probability after 12 months	− 0.5665**	0.1286	− 0.0559	1		
Median survival time	− 0.4595**	− 0.1432	− 0.1235	0.6732**	1	
Agency-employed nurse FTE ratio	0.2368	− 0.1276	0.0593	0.0598	− 0.2146	1

*AHP* Aboriginal health practitioners, *FTE* full-time equivalent

*P < 0.05, **P < 0.01

Correlations between different workforce metrics and health service characteristics were mostly statistically non-significant. Annual turnover rates, however, had a weak negative correlation with an indicator of staff supply (average number of nurses and AHPs actually working). The experienced nurse or AHP indicator had positive correlations of moderate strength with several indicators of health service size (community population size, average number of nurses and AHPs actually working and average number of FTE nurse and AHP positions on 2015 organisational chart) (Table 5).

**Table 5:** Correlations between workforce metrics for nurses and Aboriginal health practitioners 2013–2015 and health service characteristics

Health service characteristic	Annual turnover	Stability after 12 months	2 years + experienced nurse or AHP	12 month survival probability	Median survival	Agency-employed nurse FTE ratio
Distance to the nearest hospital	0.1071	− 0.1872	− 0.1074	− 0.0720	− 0.0499	0.2182
Distance to Darwin or Alice Springs	− 0.0146	− 0.2303	− 0.0156	0.0073	− 0.0419	0.2378
Community population size	− 0.2183	− 0.0587	0.3846**	0.1080	0.1255	0.1757
Average number of nurses and AHPs actually working	− 0.2929*	− 0.0270	0.5163**	0.0419	0.1232	0.0900
Average number of FTE nurse and AHP positions on 2015 organisational chart	− 0.1799	− 0.1856	0.4076**	0.0167	0.1575	0.0831

*P < 0.05; **P < 0.01

*AHP* Aboriginal health practitioners, *FTE* full-time equivalent

## Discussion

This landmark study is one of very few studies that measure turnover and retention from the perspective of a particular health service. Taking this perspective is important because it reflects what the experience of health professional continuity of care might be like from the viewpoint of consumers in a remote community. The study reveals extremely high annual turnover and poor retention for nurses and AHPs in remote NT health services. While combined nurse and AHP turnover and retention rates are less extreme when defined as either leaving remote services, they are nevertheless highly unstable and far higher than what has been reported in NT and in comparable contexts elsewhere [17, 18]. Sub-analysis of unpublished NTG DoH data by individual professional groups shows that the organisational turnover from NTG DoH was 34% for nurses (32%–36%), 22% for AHPs (13%–30%) and 33% for all staff (32%–34%) between 2013 and 2015. This is consistent with our study which found that turnover rates were significantly higher for nurses compared to AHPs. While our study was not designed to explore the underlying reasons for differences in turnover of nurses compared to AHPs, it is likely that an important effect relates to many AHPs working in communities located on their traditional land and in which they have extended family. The study also confirms anecdotal reports that agency nurses provide a substantial proportion of primary care in NT remote communities, without whose services there would undoubtedly be significant gaps in the availability of primary care services.

These findings have important implications for remote health services more generally, their patients and for policymakers. Firstly, very high turnover rates mean that health services in remote communities will need to invest considerable resources to adequately prepare and orient new staff to the health service and community [22]. Of course, there is a trade-off between investing resources in orienting new staff and investing those resources directly in providing health services for the community, particularly in an under-resourced environment. Clearly, the shorter the period of time new staff intend to stay in a remote clinic, the more the balance shifts towards investing directly in providing health services and away from providing extensive orientation, since the return on investment in orienting new staff will be small for the most short-term staff. Nevertheless, it remains important that new staff are trained to provide appropriate and culturally safe care.

Secondly, lower turnover and higher stability rates amongst AHPs compared to nurses suggest that remote workforce stability may be better supported by greater career development and employment opportunities for local Aboriginal community members to become AHPs and nurses. Increased employment of Aboriginal local community members in a range of other positions, including administrative (community workers, alcohol and other drug workers, etc.) and logistic support roles (drivers, cleaners, gardeners) may similarly help provide improved overall health workforce stability and simultaneously improve accessibility, quality of care and cultural appropriateness of health care [27].

Thirdly, high staff turnover and low stability rates are also likely to result in the already limited funding available for remote health services being substantially less than would otherwise be available because of the excess costs of recruitment, agency fees and transport, orientation and induction, housing and other higher costs for new staff and for agency staff [27]. Extremely high turnover rates may also increase risks to staff safety as constantly changing staff may be associated with decreased awareness of occupational health safety hazards [28]. Vacancies associated with staff turnover may also exacerbate safety risks for remaining resident staff, as fewer than the optimal numbers of staff are available to deliver services. High turnover and poor retention, together with high use of short-term agency staff, are also likely to limit the ability of the health service to provide high-quality care and participate meaningfully in continuous quality improvement activities [27]. Lack of stable resident primary care providers is also likely to compromise the effectiveness of visiting specialist and allied health services, since primary care providers in remote communities have a key role to identify and prioritise who needs to be seen for what and subsequently to implement and monitor any required follow-up.

For patients, high use of agency staff by health services, in conjunction with low retention and high turnover of NTG DoH-employed staff, results in relational discontinuity with their primary care providers. This is particularly important for Aboriginal patients, especially those with more serious or chronic health problems, as it means that the time needed for them to feel culturally safe and begin trusting their primary care providers is not available. As a result, patients may be less likely to access the care they need in a timely way [29]. Further, it is more likely that they will experience health encounters where the primary care provider is not adequately prepared for working in a complex cross-cultural environment. Ultimately, the quality of care that they receive is likely to be lower and their health outcomes poorer.

Some of the workforce metrics that were calculated in this analysis have not previously been reported in the published, peer-reviewed literature to describe workforce turnover and retention patterns. In small, remote health services which may have only one or two key nurses or AHPs, the health service workforce can be highly unstable and switch between periods of relative staffing stability and periods of high use of short-term staff. This study developed a new metric to indicate the number of pay periods for which a health service had at least one nurse or AHP who had at least 2 years’ clinical experience in that community. It is intended that each of the turnover or retention metrics used in this study, including the new metric, will be tested to assess their usefulness in predicting quality of primary care and potentially avoidable hospitalisations. While the usefulness of this metric is yet to be tested in other comparable contexts, our research nevertheless corroborates the use of multiple well-established workforce metrics, including annual turnover rates, annual stability rates and survival probabilities, to provide a comprehensive picture of patterns of workforce turnover and retention in remote health services. We found moderate to strongly significant correlations between annual turnover rates and other workforce metrics suggested by Russell et al. for use in rural health services [9]. Most workforce metrics, with the exception of experienced staff, were not significantly correlated with health service characteristics. This perhaps reflects that the 53 health services in the study were all at the extreme end of the spectrum of staff turnover and retention experienced in Australian health services and therefore unable to be differentiated according to community population size or distances to the nearest hospitals or to Darwin/Alice Springs.

Given the substantial policy significance of these research findings, it is important to acknowledge several limitations of the data and analysis. Firstly, data on agency-employed nurses were not available at an individual level, could not be integrated with payroll data and were not recorded in a sufficiently accurate or detailed way to enable complete capture of all agency nurses at each remote health service. The turnover rates reported are therefore underestimates, since they exclude agency nurses paid directly by agencies. Similarly, stability rates may represent overestimates of the overall patterns. Anecdotal information from health service providers confirm these assertions. The financial expenditure for labour hire costs, used to derive agency-employed nurse FTE, however, are considered to be accurate and reliable and have been used in our research to provide a comprehensive overall picture of staffing patterns in remote communities.

Secondly, it was not possible to allocate staff on the NTG DoH payroll working in a supernumerary capacity to specific health services if the cost centre covered multiple remote services. However, these comprised only a small proportion of nursing and AHP staff. Similarly, we were unable to allocate all of the aggregated agency-employed nurse FTE data to specific remote health services, as some of the cost centres covered multiple remote services. Further, our definition of agency-employed nurse excludes those agency nurses paid directly by NTG DoH, and our analysis has not attempted to specifically identify agency nurses on NTG DoH casual or temporary contracts. Our overall reported agency-employed nurse FTE ratio of 0.15 is therefore likely to be a substantial underestimate of total NTG DoH use of agency nurses. One recent report quotes a figure of 42% of remote nursing positions filled by agency-employed and NTG DoH-employed agency nurses [22]. Anecdotal evidence from nurse coordinators also indicates that between a third and a half of remote NT nursing positions are currently filled by agency nurses.

Thirdly, in focussing our analysis on nurses and AHPs, we do not capture all clinical and non-clinical staff working at remote health services. Remote health services function with the support of resident administrative staff, Aboriginal community workers, alcohol and other drugs workers, Aboriginal mental health workers, cleaners, drivers, gardeners, receptionists, community liaison officers, healthy lifestyle educators and so on. A small number of remote communities also have resident doctors for whom workforce mobility information was not available. Further, remote health clinics are often supported by visiting outreach workers providing medical, nursing and allied health services across a range of areas including public health, continuous quality improvement, health promotion, preventable chronic disease coordination and across various health care specialties. This analysis, therefore, while focused on the nurse and AHP workforce who are resident in communities, does not capture the entire spectrum of health providers working in these communities.

Nonetheless, these limitations notwithstanding, the implications of the findings of this research for policymakers are profound. In the face of such high turnover, we need robust health service models that are adequately funded, competently managed and clinical protocol driven. It is also crucial that health workforce policies are developed that effectively stabilise the remote primary care workforce and optimise workforce turnover because the benefits of continuing heavy reliance on short-term nursing staff are offset, and at times entirely negated by, substantial downsides. Stabilising the remote workforce may require different workforce models to be utilised. For example, individuals may work 1 month on, 1 month off in remote communities in shared positions. Other possible solutions include emulating medical workforce training strategies with preferential selection of rural or remote students into nursing and AHP training courses, providing vocational training based in rural and remote settings, supporting students with remote scholarships and providing recruitment and retention incentives for working in remote locations once students graduate. Another strategy may be to eliminate barriers that remote dwelling Aboriginal Australians face when entering and remaining in the health workforce. These barriers include English literacy and numeracy levels, adequacy of remuneration for AHPs, and employment conditions such as lack of subsidised housing, each of which may act as a deterrent. In the face of geographical maldistribution of doctors, community-based remote nurse practitioners may help to stabilise the workforce and improve access.

## Conclusions

This research provides rigorous empirical evidence that in remote NT communities with NTG DoH health services, turnover of nurses and Aboriginal health practitioners is extremely high, stability rates are low and substantial use is made of agency nurse services. These staffing patterns are almost certainly contributing to sub-optimal continuity of care, compromised health outcomes, poorer levels of staff safety and higher costs. To effectively address these deficiencies, it is imperative that Territory and Federal Governments invest in implementing, adequately resourcing and evaluating staffing models which effectively stabilise the remote primary care workforce as a matter of priority. The results are also important for quantifying workforce patterns in a rural or remote area, a subject for which there has been substantial national and international interest but limited research.

## Abbreviations

95%CI: 95% confidence interval
AHP: Aboriginal health practitioner
AHW: Aboriginal health worker
DoH: Department of Health
FTE: Full-time equivalent
GAS: Government accounting system
IQR: Interquartile range
NT: Northern Territory
NTER: Northern Territory emergency response
NTG: Northern Territory Government
PIPS: Personnel information and payroll systems
